# Hydrophilic catheters for intermittent catheterization and occurrence of urinary tract infections. A retrospective comparative study in patients with spinal cord Injury

**DOI:** 10.1186/s12894-024-01510-y

**Published:** 2024-06-12

**Authors:** Sajjad Ali, Omar Sufyan Khan, Amira M. Youssef, Iram Saba, Deem Alfedaih

**Affiliations:** 1 Infectious Diseases Department, Sultan bin Abdulaziz Humanitarian City, P.O.Box: 64399, Riyadh, 11536 Saudi Arabia; 2 Research and Scientific Center, Sultan bin Abdulaziz Humanitarian City, Riyadh, Saudi Arabia; 3 College of Medicine, Princess Nourah bint Abdulrahman University, Riyadh, Saudi Arabia

**Keywords:** Hydrophilic catheters, Polyvinyl chloride (PVC) catheters, Spinal cord injury (SCI), Symptomatic UTI, Bacteriuria, Pyuria

## Abstract

**Background:**

Neurogenic bladder dysfunction is a major problem for spinal cord injury (SCI) patients not only due to the risk of serious complications but also because of the impact on quality of life. The main aim of this study is to compare the rate of urinary tract infection (UTI) associated with hydrophilic-coated catheters versus uncoated polyvinyl chloride (PVC) catheters among SCI patients presenting with functional neurogenic bladder sphincter disorders.

**Methodology:**

This was a retrospective cohort study from 2005 to 2020 including adult male or female patients who have an SCI at least more than 1 month ago with neurogenic bladder dysfunction and were using intermittent catheterization (single-use hydrophilic-coated or the standard-of-care polyvinyl chloride uncoated standard catheters) at least 3 times a day to maintain bladder emptying.

**Results:**

A total of 1000 patients were selected and recruited through a stratified random sampling technique with 467 (47.60%) patients in the uncoated catheter arm and 524 (52.60%) in the coated catheter groups. The three outcome measures, namely: symptomatic UTI, Bacteriuria, and pyuria were significantly higher in the group using uncoated polyvinyl chloride (PVC) catheters compared to hydrophilic-coated catheters at the rate of 79.60% vs.46.60%, 81.10% vs. 64.69, and 53.57% versus 41.79% respectively. Males, elder patients, longer duration, and severity of SCI were associated with increased risk of symptomatic UTI.

**Conclusions:**

The results indicate a beneficial effect regarding clinical UTI when using hydrophilic-coated catheters in terms of fewer cases of symptomatic UTI. Bacteriuria is inevitable in patients with long-term catheterization, however, treatment should not be started unless the clinical symptoms exist. More attention should be given to the high-risk group for symptomatic UTIs.

**Supplementary Information:**

The online version contains supplementary material available at 10.1186/s12894-024-01510-y.

## Introduction

Spinal cord dysfunction, due to spinal cord injury (SCI), is one of the major causes of neurogenic bladder (NB) [1]. One of the main treatment strategies to overcome this problem is urinary tract catheterization. Prolonged indwelling catheters however often cause complications such as urinary tract infections (UTI), bladder stones, bladder cancer, and urethral injury or fistula. Substituting with clean intermittent catheterization (CIC) may reduce such complications, but CIC increases the risk of bacteriuria in patients with NB by introducing urethral microorganisms into the bladder during repeated catheterizations [2].

CIC was originally performed with polyvinyl chloride (PVC) catheters, however, to reduce catheter-related complications, a low-friction hydrophilic catheter was introduced in 1983 [3]. Hydrophilic catheters coated along the entire length with a hydrophilic substance produce a smooth, slippery surface with 10 to 15 folds less friction than a standard polyvinyl catheter [4].

Some international studies and reviews have shown lower UTIs among patients using hydrophilic coated catheters compared to users of uncoated catheters [5, 6]. There are two proposed advantages of hydrophilic coated catheters over uncoated catheters; mainly reducing urethral irritation and injury, in addition to lowering the incidence of symptomatic UTI [7]. Although previous studies and reviews have shown conflicting results, studies in favor of the hydrophilic-coated catheter in reducing UTIs compared to non-coated catheters are limited [8, 9].

Therefore, this study aims to explore the experience of a tertiary center that hosts a large number of patients with SCI for a prolonged period, by reviewing the patients’ files for the last 15 years, evaluating the rate of UTI among patients who had used hydrophilic-coated catheters versus uncoated PVC catheters during that period. Part of the study is also to assess different risk factors values in precipitating UTIs.

## Methodology

### Study design and setting

This retrospective hospital-based study was conducted during the period from January 1st to June 30, 2022, at Sultan bin Abdulaziz Humanitarian City (SBAHC) looking at patients with SCI who required CIC and who were hospitalized for rehabilitation (typically 4–6 weeks). Chart review through the health information system (HIS) for the period from January 2005 to December 2020 revealed 10,000 patients, a total of 2000 patients were selected through a systematic random sampling technique, where every fifth patient was selected and then those patients were subjected to the inclusion and exclusion criteria. Patients aged ≥ 18 years regardless of their gender with neurogenic bladder and performing more than three intermittent catheterizations per day were included. The exclusion criteria included any patients with organ failure, vesico reflux, and being subjected to invasive urology procedures. Patients who had symptoms of UTI on admission or were under treatment with prophylactic antibiotics to prevent UTI or had incomplete data were also excluded. The eligible patients were stratified into two groups; the polyvinyl chloride uncoated catheter users and hydrophilic coated catheter users and a random sample was selected from each group.

**Fig. 1:**
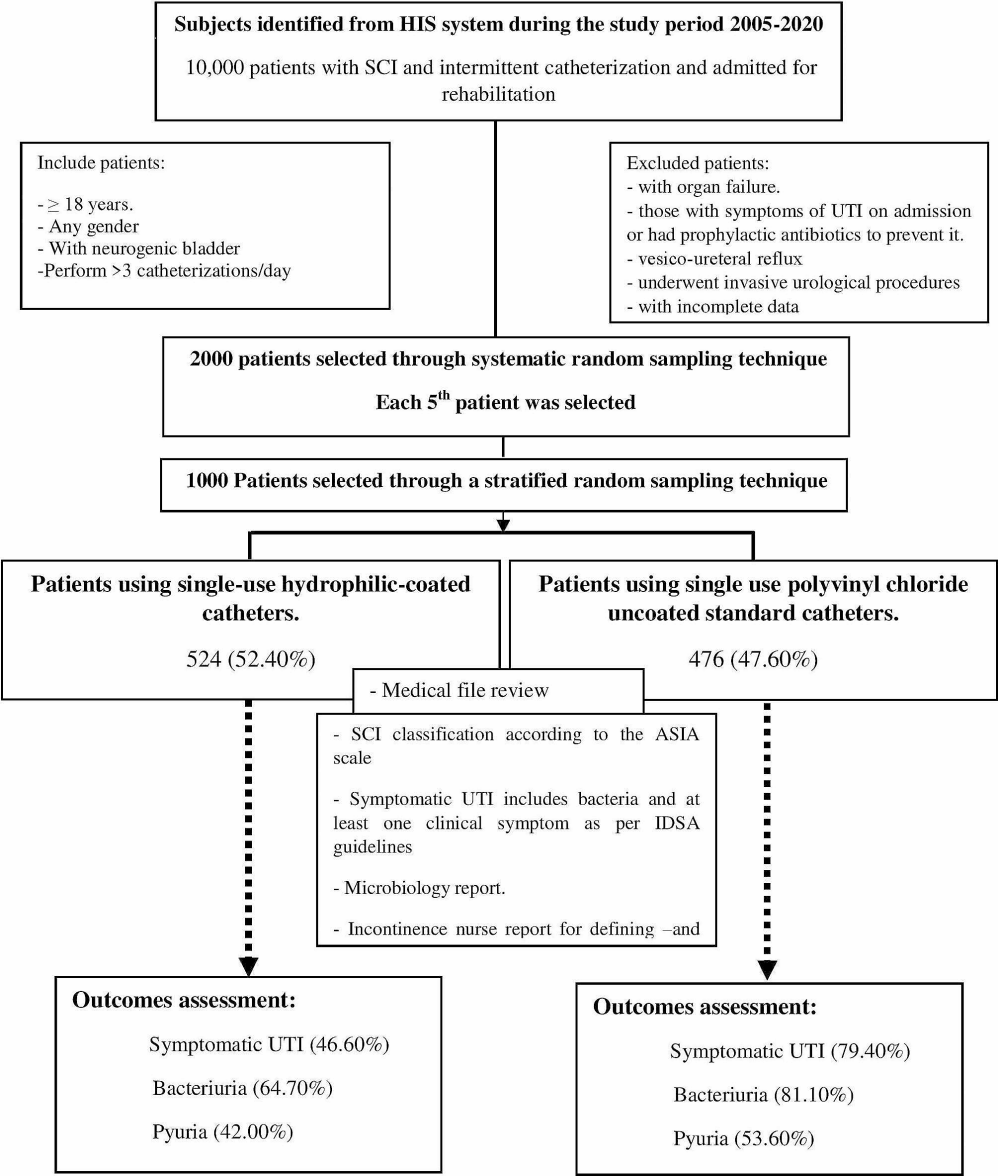
Study flow chart. *Note* IDSA: Infectious Diseases Society of America

As shown in Fig. 1, the sample selection was concluded when 1000 patients were recruited among the sample of 2000 patients. This comprised 524 patients (52.4%) using hydrophilic coated catheters, and 476 patients (47.6%) using polyvinyl chloride uncoated catheters at the time of admission. The two groups were classified regarding their spinal injury using the American Spinal Injury Association (ASIA) impairment scale into four groups; A: no motor or sensory function is preserved in the sacral segments S4–S5, B: sensory function preserved but not motor function is preserved below the neurological level and includes the sacral segments S4–S5, C: motor function is preserved below the neurological level, and more than half of key muscles below the neurological level have a muscle grade less than 3, D: motor function is preserved below the neurological level, and at least half of key muscles below the neurological level have a muscle grade of 3 or more [10].

### Exposure

The exposure was the use of intermittent catheterization type, where patients from the PVC non-coated catheter arm were collected during the period from 2005 to 2010, where this type of catheter was exclusively used in the hospital, while data related to the hydrophilic catheter users collected from 2011 to 2020 when this catheter was introduced to replace the previous type.

### Outcome measures

As per the International Clinical Practice Guidelines from the Infectious Diseases Society of America (IDSA) guidelines [11], catheter-associated asymptomatic bacteriuria was defined as having at least 10^5 colony-forming units (CFU) per ml of a single organism in the urine of a non-catheterized patient. Pyuria was defined as having positive leukocyte esterase on urine dipstick or ≥ 10 white blood cells per high-powered field (WBCs/hpf) on urine microscopy. Symptomatic UTI was defined as having a bacterial colony count of at least 10^5 colony-forming units and at least one clinical symptom, such as fever, chills, cloudy urine, increased spasticity, or malaise and was reported as yes, or no after evaluating the criteria for every single case by the research physician, any recurrent UTI events were nor reported or included in the analysis. Positive urine culture in the absence of clinical symptoms was not considered a UTI [11].

### Study variables

For both groups, socio-demographic data including age, and gender were collected using a predesigned case report form (CRF). Additionally, data related to SCI including the ASIA impairment scale, type of injury, and type of implant were also collected. Past clinical history of the patient was reported including comorbid conditions and drug history as well as the type of catheter used. All the patients’ microbiology reports were collected from a microbiology laboratory that included bacterial type and strain. The incontinence nurse report was used to document the catheter type and UTI symptoms and the daily frequency of intermittent catheterization. UTI-related data were also collected including UTI symptoms, urinalysis, urine microscopy, urine culture, and susceptibility testing.

### Statistical analysis

Prior to analysis, variable distribution was tested to ensure whether the assumption of normality was met. Qualitative variables were described in terms of frequency and percentage. Quantitative variables were described by the number of observed values with mean and standard deviation. Continuous measures across different groups were assessed using a Student’s t-test and the chi-square test was used to compare categorical variables. A p-value of < 0.05 was accepted as the level of significance. Logistic regression analyses were used to estimate odds ratios (ORs) and 95% confidence intervals (95% CI). The rates of outcome measures were assessed according to different confounding factors including age, gender, and level of ASIA scale. Univariate analysis was used to assess the factors associated with increased risk of outcome measures and was expressed as odds ratio and 95% confidence interval (CI). The multivariate regression analysis was used to assess the association between a dependent variable (i.e. symptomatic UTI, pyuria, and bacteriuria) and more than one independent variable including age, gender, duration of SCI, ASIA scale, and the number of complications.

## Results

The current study included a total of 1000 patients with SCI comprised of 87.2% males and 12.8% females with a mean age of 34.07 ± 13.19 years and a mean duration of SCI of 5.95 ± 5.84 years. This study revealed the advantage of the hydrophilic coated catheter over the PVC uncoated catheter. This difference was clearly shown with a low proportion of symptomatic UTI (46.6% versus 79.4%), bacteriuria (64.69% versus 81.09%), and pyuria (41.79% versus 53.57%) among the patients using the hydrophilic coated catheter compared to those using the PVC uncoated catheters. There was no significant difference between the two studied cohorts regarding age, gender, and co-morbidities as shown in Table 1.

**Table 1:** Baseline sociodemographic and clinical characteristics of the studied cohort according to the type of catheter

Variable	Total *n* (%)	Polyvinyl chloride uncoated catheters *n* (%)	Hydrophilic-coated catheters. *n* (%)	*P*-value
Number	1000 (100)	476 (47.65)	524 (52.4)	
Age mean age (± SD)	34.07 (13.19)	33.66 (14.12)	34.44 (12.32)	0.351
≤ 25 years	294 (29.4)	171 (35.92)	123 (23.47)	**0.001**
26–35 years	381 (38.1)	155 (32.56)	226 (43.13)	**0.002**
> 35 years	325 (32.5)	150 (31.51)	175 (33.40)	0.607
Gender Male	872 (87.2)	417 (87.61)	455 (86.84)	0.715
Female	128 (12.8)	59 (12.39)	69 (13.16)	0.715
SCI mean duration (± SD)	5.95 (5.84)	4.22 (4.19)	7.49 (6.64)	**< 0.0001**
1–2 years	327 (32.7)	196 (41.18)	131 (25.00)	**< 0.0001**
3–5 years	337 (33.7)	180 (37.82)	157 (29.96)	**0.0312**
> 5 years	336 (33.6)	100 (27.01)	236 (45.04)	**< 0.0001**
ASIA scale A	601 (60.1)	215 (45.16)	386 (73.66)	**< 0.0001**
B	192 (19.2)	115 (24.15)	77 (14.69)	**0.0001**
C	147 (14.7)	100 (21.01)	47 (8.96)	**< 0.0001**
D	60 (6.0)	46 (9.67)	14 (2.67)	**< 0.0001**
Number of comorbidities 1	401 (40.1)	198 (41.5)	203 (38.74)	**0.384**
2	60 (6.0)	30 (6.30)	30 (5.72)	0.699
≥ 3	69 (6.9)	9 (1.89)	60 (11.45)	**< 0.0001**

ASIA: The American Spinal Injury Association (ASIA) Impairment Scale; SCI: Spinal Cord Injury

The bold vlaue are the significant *p*-values

There was a significantly lower proportion of UTI symptoms in terms of fever, spasticity, and cloudy urine among patients using the hydrophilic coated catheter compared with patients using PVC catheters. Additionally, patients using hydrophilic coated catheters had a significantly lower proportion of positive nitrate using urinary dipstick test and bacteriuria as shown in Table 2. As shown in Appendix S1, in supplementary material, the proportion of symptomatic UTI was significantly higher among the patients who were using PVC uncoated catheters across different age groups and the two genders, ASIA scale levels, SCI duration categories, and different numbers of co-morbidities, *p* < 0.001, except for ASIA scale D (*p* < 0.175) and more than three complications (*p* = 0.05).

**Table 2:** Urinalysis and clinical symptoms of urinary tract infection among the studied cohort according to the type of catheter

Variable	Total *n* (%)	Polyvinyl chloride uncoated catheters *n* (%)	Hydrophilic-coated catheters *n* (%)	*P*-value
Number	1000 (100)	476 (47.65)	524 (52.4)	
Urine dipstick test Leukocyte esterase	564 (56.4)	255 (53.57)	309 (58.97)	0.098
Urine dipstick test Nitrite	640 (64.0)	337 (70.79)	303 (57.82)	**< 0.0001**
Urine microscopy for WBCs	629 (62.9)	287 (60.30)	342 (65.27)	0.077
Bacteriuria	725 (72.5)	386 (81.09)	339 (64.69)	**< 0.0001**
Symptoms Fever	542 (54.2)	331 (69.54)	215 (41.03)	**< 0.0001**
Incontinence	353 (35.3)	171 (35.93)	182 (34.73)	0.7389
Spasticity	304 (30.4)	254 (53.36)	50 (9.54)	**< 0.0001**
Cloudy urine	198 (19.8)	63 (13.23)	136 (25.95)	**0.0014**

WBCs: White Blood Cells

The bold vlaue are the significant *p*-values

**Fig. 2:**
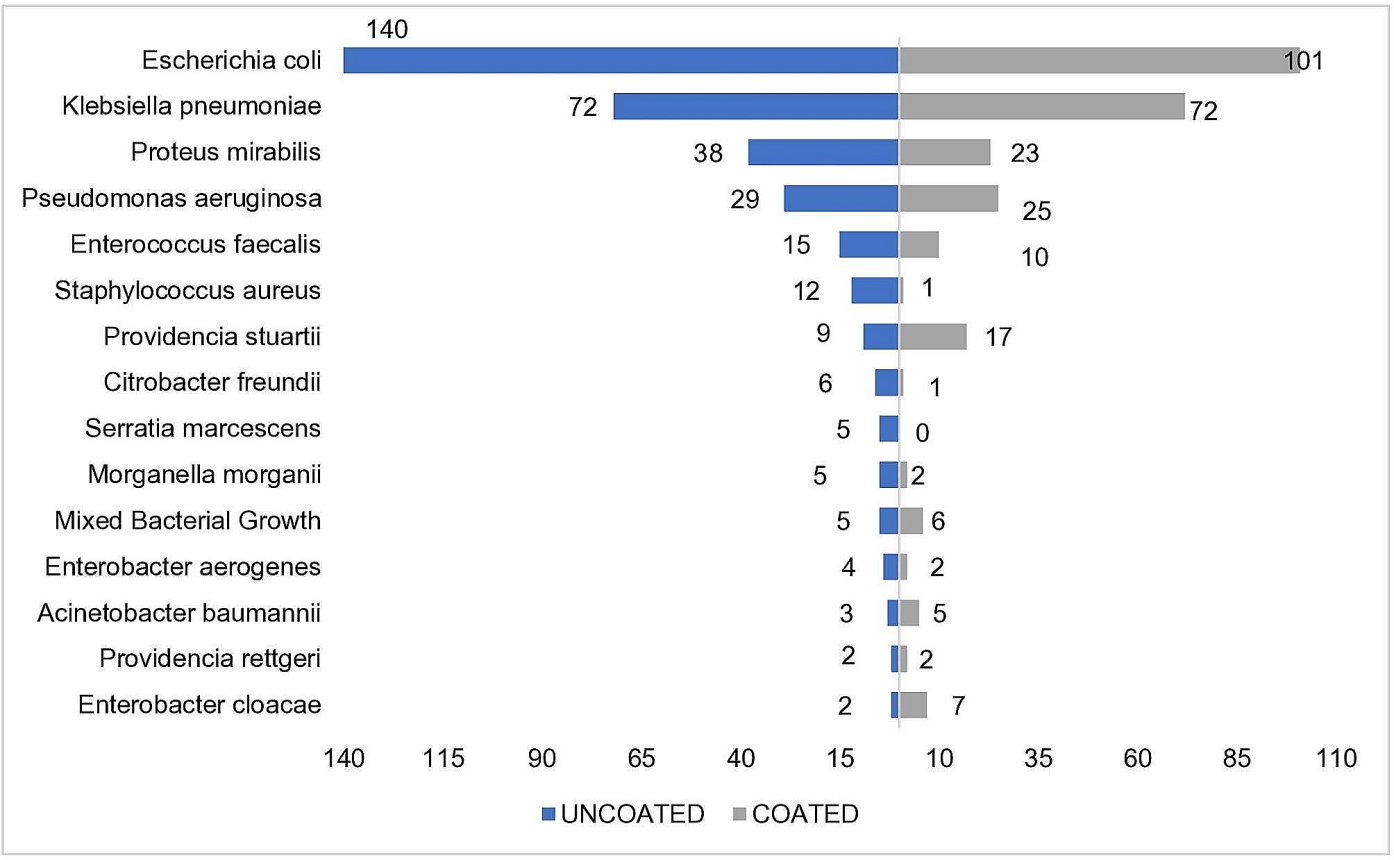
The number of different isolated bacterial species from the two cohorts

When looking at the culture results, the patients using PVC uncoated catheters had significantly higher rates of bacterial species compared to the ones using hydrophilic coated catheters, at 72.89% versus 52.29% respectively. *Escherichia coli* was the most commonly isolated organism for patients using either coated or uncoated catheters, although it was more common among patients using uncoated catheters, while *Klebsiella pneumonia* was the second most common organism in the two groups of patients with no difference in frequency between the coated and the uncoated catheters users. *Proteus mirabilis*, *Pseudomonas aeruginosa*, and *Enterococcus faecalis*, all were isolated more frequently among patients using uncoated catheters. Staphylococcus aureus and *Providencia stuartii* have been noted to be more prominent in the coated catheter cohort. The other isolated bacteria were rare and demonstrated variability among both coated and uncoated catheter groups, please see Fig. 2.

**Fig. 3:**
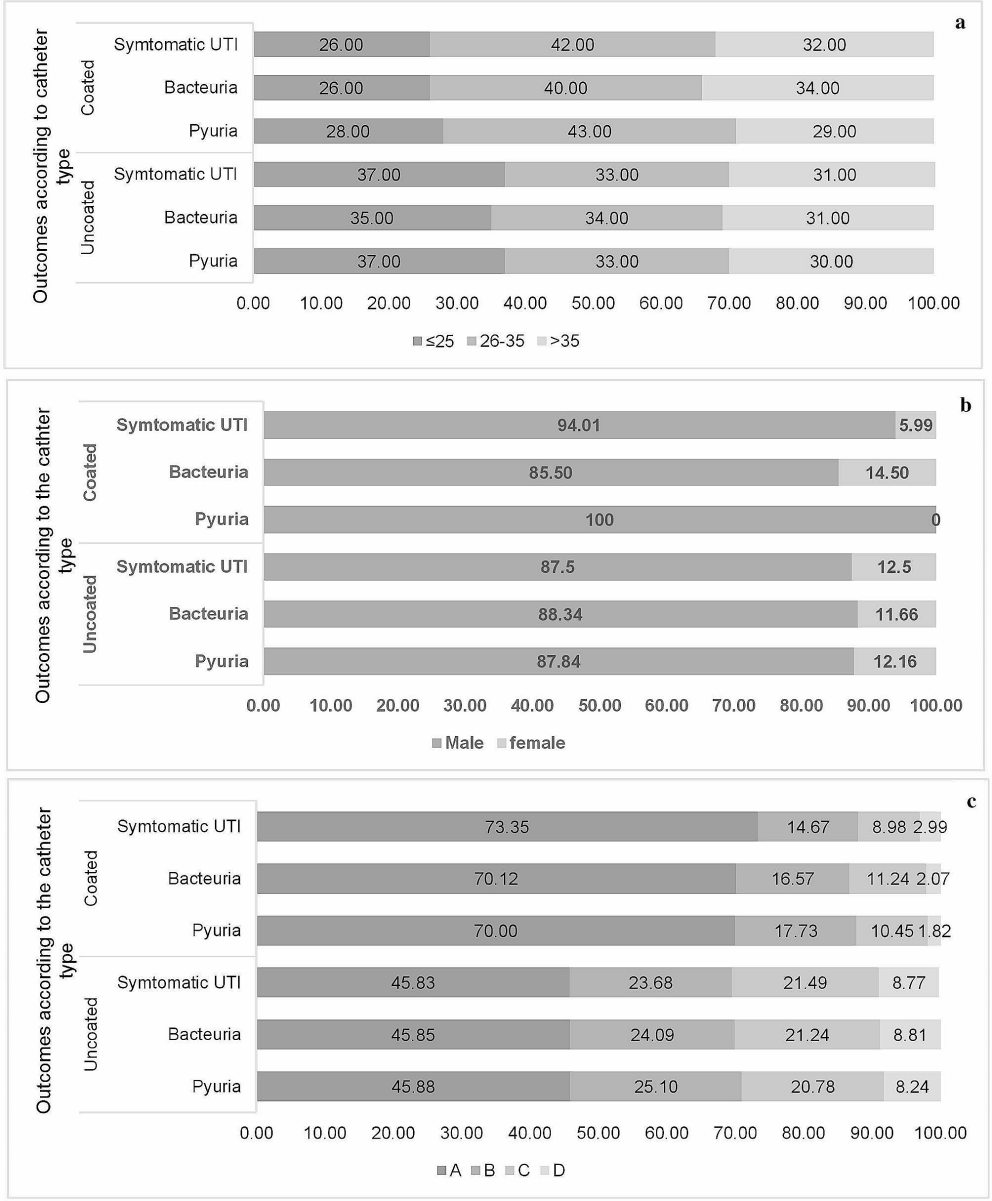
Distribution of symptomatic urinary tract infection (UTI), bacteriuria, and pyuria by age (**a**); gender (**b**); and ASIA scale (**c**) among the study cohort according to the type of catheter

When considering the age groups ≥ 25 years, 26–35 years and > 35 years, patients using coated catheters had less symptomatic UTI, bacteriuria, and pyuria compared to those who are using PVC uncoated catheters. Patients with symptomatic UTI, pyuria, and bacteriuria and using hydrophilic coated catheters were having significantly higher rates of males compared with PVC uncoated catheter users. Additionally, there was a higher frequency of class A and D among patients with symptomatic UTI, pyuria, and bacteriuria and using hydrophilic coated catheters, compared to the PVC uncoated catheters patients. On the other hand, patients using PVC uncoated catheters had higher rates of ASIA scale class C in three reported outcomes compared to the hydrophilic coated catheter patients as shown in Fig. 3.

As shown in Table 3, the OR of developing symptomatic UTI was more than 12 times higher among the patients who used the PVC uncoated catheters compared to those who were using the hydrophilic coated catheters in the univariate analysis, OR [95% CI] (12.97 [8.0–21.0], *P* < 0.001). This association remained after conducting the multivariate analysis indicating that the association was independent of age, gender, duration of spinal cord injury, ASIA scale, and the number of comorbidities. This independent association was also observed between the uncoated catheter use and both pyuria and bacteriuria but to a lower extent with OR [95% CI] for the univariate analysis at 1.59 [1.2-2.0], *p* < 0.001, and 2.34 [1.7–3.1], *p* < 0.001 respectively.

**Table 3:** Univariate and multivariate logistic regression analysis of the outcome measures among patients using uncoated hydrophilic catheters versus patients using coated hydrophilic catheters

Outcomes	Uncoated vs. Coated catheter (univariate analysis)		Uncoated vs. Coated catheter (multi-variate analysis)					
	OR (95% CI)	*p*-value	Model ‘a’		Model ‘b’		Model ‘c’	
			OR (95% CI)	*p*-value	OR (95% CI)	p-value	OR (95% CI)	p-value
Symptomatic UTI	12.97 (8.0–21.0)	< 0.001	12.95 (8.0–20.9)	< 0.001	13.65 (8.3–22.3)	< 0.001	16.14 (9.4–27.2)	< 0.001
Pyuria	1.59 (1.2–2.0)	< 0.001	1.58 (1.2–2.0)	< 0.001	1.62 (1.2–2.1)	< 0.001	1.62 (1.2–2.1)	0.001
Bacteriuria	2.34 (1.7–3.1)	< 0.001	2.34 (1.8–3.1)	< 0.001	2.39 (1.8–3.2)	< 0.001	2.33 (1.7–3.2)	< 0.001
New Fever	3.27 (2.5–4.2)	< 0.001	3.26 (2.5–4.2)	< 0.001	3.48 (2.6–4.6)	< 0.001	8.96 (5.3–15.2)	< 0.001
Incontinence	1.05 (0.8–1.4)	0.69	1.05 (0.7–1.5)	0.79	1.04 (0.8–1.4)	0.78	0.86 (0.6–1.2)	0.32
Spasticity	10.85 (7.7–15.3)	< 0.001	10.83 (7.7–15.3)	< 0.001	10.78 (7.6–15.4)	< 0.001	11.75 (8.0–17.2)	< 0.001

UTI: Urinary Tract Infection; CI: Confidence Interval; OR: Odds Ratio

The data has been generated by logistic regression analysis where catheterization being uncoated vs. coated was a dependent variable. Model ‘a’, ‘b’, and ‘c’ represents ORs adjusted for age + gender, age + gender + duration, and age + gender + SCI duration + ASIA scale + the number of comorbidities respectively

When performing univariate logistic regression analysis, male gender, and ASIA scale level C or higher were associated with an increased risk of symptomatic UTI in the studied patients, where there was 84% higher OR for symptomatic UTI among men compared to women (OR = 1.84; 95% CI: 1.3–2.7), while ASIA scale ≥ C was associated with a 31–41% higher OR for symptomatic UTI. However, in the multivariate logistic regression analysis, only the male gender remained a significant risk factor. On the other hand, age ≥ 25 years and SCI duration ≥ 10 years were associated with reduced risk of symptomatic UTI, with this association being independent of gender, and the number of complications as shown in Table 4. As shown in Appendix S2, supplementary material, only having one complication was independently associated with an increased risk of pyuria, OR [95% CI], 1.4 (1.1–1.9), *P* = 0.011. None of the studied risk factors were associated with an increased risk of bacteriuria, except for ASIA scale ≥ C which was independently associated with a 40% increased risk of bacteriuria, as shown in Appendix S3, supplementary material.

**Table 4:** Univariate and multivariate logistic regression analysis for factors associated with the development of symptomatic urinary tract infection (UTI) among the study cohort regardless of catheter used

Variables	Symptomatic UTI (univariate analysis)		Symptomatic UTI (multi-variate analysis)					
	OR (95% CI)	*P*	Model ‘a’		Model ‘b’		Model ‘c’	
			OR (95% CI)	*P*	OR (95% CI)	*P*	OR (95% CI)	*P*
Male gender^α^	1.84 (1.3–2.7)	0.001	1.69 (1.2–2.5)	0.006	-	-	1.58 (1.1–2.3)^*^	0.019
Age ≥ 25 years^β^	0.47 (0.3–0.6)	< 0.001	-	-	0.48 (0.4–0.7)^*^	< 0.001	0.51 (0.4–0.7)^*^	< 0.001
SCI duration ≥ 10 years^¥^	0.61 (0.4–0.8)	0.002	0.64 (0.5–0.9)	0.006	0.66 (0.5–0.9)	0.009	-	-
ASIA scale C&D^£^	1.81 (1.1–2.7)	0.006	1.41 (1.0–2.0)	0.044	1.38 (1.0–1.9)	0.056	1.34 (1.0–1.9)	0.087
One chronic complication^$^	1.18 (0.9–1.6)	0.240	1.13 (0.9–1.5)	0.393	1.11 (0.8–1.5)	0.478	1.10 (0.8–1.5)	0.509
Two chronic complications^$^	1.47 (0.8–2.6)	0.194	1.44 (0.8–2.6)	0.233	1.41 (0.8–2.6)	0.267	1.42 (0.8–2.6)	0.273
≥Three complications^$^	0.71 (0.4–1.2)	0.178	0.69 (0.4–1.2)	0.156	0.65 (0.4–1.1)	0.109	0.68 (0.4–1.2)	0.159

ASIA: The American Spinal Injury Association (ASIA) Impairment Scale; CI: Confidence Interval; OR: Odds Ratio; SCI: Spinal Cord Injury. Note The data has been generated by logistic regression analysis where symptomatic UTI ‘yes’ vs. ‘no’ was a dependent variable. Model ‘a’, ‘b’, and ‘c’ represents OR’s adjusted with age, age + gender, and age + gender + duration in years respectively. * depicts the independent variable has been excluded from the model. ^α^ depicts the odds ratio with female gender as reference. ^β^ depicts the odds ratio with age < 25 years as reference. ^¥^ depicts the odds ratio with SCI duration < 10 years. ^£^ depicts the odds ratio with ASIA scale A&B. ^$^ depicts the independent variables one, two, and ≥ chronic complications have been checked in comparison to ones with no chronic complications

## Discussion

The current study has highlighted the advantage of using hydrophilic coated catheters compared to PVC uncoated catheters in terms of lower proportions of symptomatic UTI, pyuria, and bacteriuria amg a cohort of SCI in a rehabilitation setting. CIC is beneficial for the long-term care of patients with neurogenic bladders. Low intravesical pressure and frequent bladder voiding allow for the preservation of a balanced bladder. The incidence of urinary tract degeneration in patients with SCI was significantly reduced in numerous studies by long-term clean intermittent catheterization [12]. However, other studies that reported high withdrawal rates and high incidence rates of complications indicate that clean intermittent catheterization is not the ultimate solution. Adding a hydrophilic coating was proven to reduce the coefficient of friction of standard PVC catheters by up to 90% [13], which was associated with less trauma, reduced rate of strictures, increased patient satisfaction, and potentially reduced rate of UTIs.

In the current study, the demographic characteristics were similar in the two study groups with no significant difference in terms of mean age, and the male-to-female ratio. The result of the current study shows that using hydrophilic-coated catheters results in fewer rates of symptomatic UTIs when compared to using conventional non-hydrophilic catheters which is in accordance with previous studies that showed trends in favor of using hydrophilic-coated catheters [4, 12, 14, 15]. The rate of symptomatic UTIs was almost two times higher in the patients using PVC uncoated catheters compared with the patients using hydrophilic coated catheters (46.6% versus 79.4%) which is similar to the observation of De Ridder et al., with rates of 64% versus 82% respectively [16]. Additionally, this is also in line with the findings of a systematic review that has shown that the UTI incidence rate was 49.6% in patients using hydrophilic catheters and 72.0% for patients using a non-hydrophilic catheter [17].

Bacteriuria is unavoidable in patients with long-term catheterization, where the rate of bacteriuria among the patients using PVC uncoated catheters in this study was 81.15% which was significantly higher among the ones using coated catheters. This was also observed by Perkash et al., where the bacteriuria rate was reported to be 86% [18]. The rate of pyuria was also significantly higher in the uncoated catheter group. which is similar to the findings of Jeong et al. [19]., but does not correspond to the findings of De Ridder et al., who reported a significant difference in the rate of symptomatic UTI only but no significant different in terms of bacteriuria or pyuria [16].

It is worth mentioning that the IDSA and AUA guidelines recommend against screening for or treatment of asymptomatic bacteriuria in those with SCI and short or long-term indwelling catheters [20, 21]. However, the signs and symptoms of UTI in those with SCI or other neurologic pathology are often subtle and different from those without neurologic pathology, therefore, clinicians should carefully assess signs and symptoms in this population.

The majority of organisms that are isolated from the urine culture of UTI patients are either from the patient’s own colonic flora or exogenous organisms from the hospital environment. Exogenous organisms may colonize catheter equipment if it is transferred via the hands of health care personnel. Different bacterial species were isolated from the urine culture of the two studied groups of patients with *Escherichia coli* and *Klebsiella pneumonia* being the most frequently isolated pathogen, which is in line with the finding of Ploypetch et al. [22]. Since nosocomial-symptomatic UTIs can prolong rehabilitation time missed therapy sessions, also necessitate lengthy antibiotic treatment, which can considerably affect the health and economic value [23]. The current study suggests that using the hydrophilic-coated catheter could minimize the incidence of UTI and its related complications.

The current study has demonstrated that the male gender, and ASIA scale C are associated with an increased risk of symptomatic UTI, while older age and long duration of SCI were associated with reduced risk of symptomatic UTI. This is the same observation of Mukai et al. [24]. In the current study, age ≥ 25 years was associated with an independent significantly reduced risk of developing symptomatic UTI, which contradicts the findings of other studies that reported advanced age as a risk factor for the catheter induced UTI [25, 26]. Our findings indicated that patients with ASIA class C or D cases were substantially associated with UTI occurrence. This result cannot be explained scientifically stringently at the moment. Further independent studies should therefore first verify these results.

We would like also to emphasize the limitations of this study. First, this study is retrospective, which is not the proper setting to assess causality. However, there are several limitations to conducting a prospective study among the SCI cohort due to several reasons including; the extensive nature of physical injuries of persons with acute SCI that could lead to termination of the patient’s participation in a prospective study and challenges in reporting the adverse events among such patients after the discharge. The second limitation of the current study is that it did not report other adverse events such as hematuria and urethral trauma, however, a recent systematic review elaborated that there is no significant difference between coated and un-coated catheters in the rate of those complications [22]. The third limitation could be the possibility of having changes in the guidelines during the different study periods (2005 to 2010 and 2011 to 2020). Additionally, the recurrence rate was not reported, however, it was not within the scope of the study. One more limitation to be highlighted is that some confounding factors that might have affected the results were not addressed such as the overall bladder management that was provided to the patients in the hydrophilic coated catheter group. The main strength of this study is that it assessed the rate of symptomatic UTI among a large cohort of patients in a rehabilitation setting using a robust methodology. The second strength was the use of the most recent guidelines for defining the symptomatic UTI.

## Conclusion

In conclusion, the use of intermittent catheterization has improved the care of these patients, but infections still arise. The hydrophilic-coated catheters had superior beneficial clinical effects compared to the PVC uncoated catheters, where there were significantly fewer patients who experienced symptomatic UTIs in the hydrophilic-coated catheters group compared to the uncoated catheter group with almost 50% the patients using the hydrophilic-coated catheter group were free from symptomatic UTIs. Such findings may greatly impact the quality of life of SCI patients by reducing their risk of developing symptomatic UTIs. Our data demonstrated that male gender, old age patients, patients with longer SCI duration, and ASIA impairment scale C or more severe were associated with an increased risk of developing symptomatic UTI in SCI patients. Therefore, more attention could be given to this group while assessing and monitoring for UTI symptoms. Even though bacteriuria is inevitable in patients with long-term catheterization, however, treatment should not be started unless the clinical symptoms exist.

### Electronic supplementary material

Below is the link to the electronic supplementary material.

Supplementary Material 1

## Data Availability

Data is provided within the manuscript or supplementary information files.
