# Supplementary material for: Hydrophilic catheters for intermittent catheterization and occurrence of urinary tract infections. A retrospective comparative study in patients with spinal cord Injury
Source: BMC Urol. 2024 Jun 12;24:122. doi: 10.1186/s12894-024-01510-y (PMC11167762; doi:10.1186/s12894-024-01510-y)
Supplement: Supplementary file 1 — Supplementary Material 1 [file 12894_2024_1510_MOESM1_ESM.docx]

**Appendix 1. The proportion of patients with pyuria, bacteriuria, and symptomatic, urinary tract infection (UTI) according to different demographic and clinical characteristics.**

| Variables | | Total | | Pyuria | | | Bacteriuria | | | Symptomatic UTI | | p-value |
| --- | --- | --- | --- | --- | --- | --- | --- | --- | --- | --- | --- | --- |
|  |  | Uncoated catheter  (n) | Coated  catheter  (n) | Uncoated  catheter  n (%) | Coated  catheter  n (%) | p-value | Uncoated  catheter  n (%) | Coated  catheter  n (%) | p-value | Uncoated  Catheter  n (%) | Coated  catheter  n (%) |  |
| Age (Years) | ≤25 | 171 | 123 | 95 (55.56) | 62 (50.41) | 0.383 | 136 (79.53) | 88 (71.54) | 0.113 | 167 (97.66) | 86 (69.92) | **<0.001** |
|  | 26-35 | 155 | 226 | 84 (54.19) | 95 (42.04) | **0.019** | 130 (83.87) | 136 (60.18) | **<0.001** | 149(96.13) | 140 (61.95) | **<0.001** |
|  | >35 | 150 | 175 | 76 (50.67) | 63 (36.00) | **0.007** | 120 (80.00) | 11 4(65.14) | **0.003** | 140 (93.33) | 108 (61.95) | **<0.001** |
| Gender | Male | 417 | 455 | 224(53.72) | 220 (48.35) | 0.113 | 341 (81.77) | 289 (63.52) | **<0.001** | 399 (95.68) | 314 (69.01) | **<0.001** |
|  | Female | 59 | 69 | 31 (52.54) | 224 (53.72) | 0.894 | 45 (76.27) | 49 (71.01) | 0.503 | 57 (96.61) | 20 (28.99) | **<0.001** |
| ASIA scale | A | 215 | 386 | 117 (54.42) | 154 (39.9) | **0.006** | 177 (82.33) | 237 (61.4) | **<0.001** | 209 (97.21) | 245 (63.47) | **<0.001** |
|  | B | 115 | 77 | 64 (55.65) | 39 (50.65) | 0.49 | 93 (80.87) | 56 (72.73) | 0.186 | 108 (93.91) | 49 (63.64) | **<0.001** |
|  | C | 100 | 47 | 53 (53.0) | 23 (48.94) | 0.647 | 82 (82.0) | 38 (80.85) | 0.860 | 98 (98.0) | 30 (63.83) | **<0.001** |
|  | D | 46 | 14 | 21 (45.65) | 4 (28.57) | 0.234 | 34 (73.91) | 7 (50.00) | 0.09 | 40(86.96) | 10 (71.43) | <0.175 |
| SCI duration (Years) | 1-2 | 196 | 131 | 106 (54.08) | 50 (38.46) | **0.005** | 165 (84.18) | 85 (64.88) | **0.000** | 156 (79.59) | 69 (35.20) | **< 0.000** |
|  | 3-5 | 180 | 157 | 100 (55.55) | 67 (42.67) | **0.018** | 147 (81.66) | 98 (62.42) | **0.000** | 143 (79.44) | 78 (49.68) | **< 0.000** |
|  | >5 | 100 | 236 | 49 (49.0) | 103 (43.64) | 0.367 | 74 (74.00) | 156 (66.10) | 0.154 | 79 (79.0) | 97 (41.10) | **< 0.000** |
| No of comorbidities | 1 | 198 | 203 | 111(56.06) | 100 (49.26) | 0.173 | 166 (83.84) | 135(66.5) | **0.000** | 197 (99.49) | 192 (94.58) | **0.004** |
|  | 2 | 30 | 30 | 20 (66.67) | 12 (40.00) | **0.040** | 22 (73.33) | 18 (60. 0) | 0.277 | 29 (96.67) | 20 (66.67) | **0.002** |
|  | >3 | 9 | 60 | 2 (22.22) | 25 (41.67) | 0.268 | 8 (88.89) | 39 (65.00) | 0.154 | 7 (77.78) | 26 (43.33) | 0.055 |

ASIA: The American Spinal Injury Association (ASIA) Impairment Scale; SCI: spinal cord injury.

**Appendix 2. Univariate and multivariate analysis for factors associated with the development of pyuria in the studied cohort.**

| Variables | Pyuria (univariate analysis) | | Pyuria (multi-variate analysis) | | | | | |
| --- | --- | --- | --- | --- | --- | --- | --- | --- |
|  | OR (95% CI) | P | Model ‘a’ | | Model ‘b’ | | Model ‘c’ | |
|  |  |  | OR (95% CI) | P | OR (95% CI) | P | OR (95% CI) | P |
| Male gender ^α^ | 1.2 (0.8 -1.7) | 0.272 | 1.1 (0.8-1.7) * | 0.390 | 1.1 (0.8-1.7) * | 0.412 | 1.1 (0.8-1.7) * | 0.408 |
| Age ≥ 25 years ^β^ | 0.8 (0.6 – 1.11) | 0.246 | 0.8 (0.7 – 1.1) * | 0.286 | 0.8 (0.7 – 1.1) * | 0.317 | 0.8 (0.7 – 1.1) * | 0.307 |
| SCI duration ≥ 10 years^¥^ | 0.8 (0.6 - 1.1) | 0.393 | 0.9 (0.7 – 1.2) | 0.532 | - | - | 0.9 (0.7 – 1.2) * | 0.530 |
| ASIA scale ≥ C^£^ | 0.9 (0.7 - 1.3) | 0.959 | 1.0 (0.7 – 1.4) | 0.950 | 1.0 (0.7 – 1.4) | 0.967 | - | - |
| One chronic complication^$^ | 1.4 (1.1 - 1.8) | **0.007** | 1.4 (1.1 – 1.8) | **0.015** | 1.4 (1.1 – 1.8) | **0.015** | 1.4 (1.1 – 1.9) | **0.011** |
| Two chronic complications^$^ | 1.5 (0.8 - 2.6) | 0.121 | 1.5 (0.9 – 2.6) | 0.142 | 1.5 (0.9 – 2.6) | 0.139 | 1.5 (0.9 – 2.7) | 0.385 |
| ≥Three complications^$^ | 0.8 (0.4 - 1.3) | 0.491 | 0.8 (0.5 – 1.4) | 0.436 | 0.8 (0.5 – 1.4) | 0.451 | 0.8 (0.5 – 1.4) | 0.422 |

ASIA: The American Spinal Injury Association (ASIA) Impairment Scale; SCI: spinal cord injury.

Note: The data has been generated by logistic regression analysis where pyuria and bacteriuria dare dependent variables. Model ‘a’, ‘b’, and ‘c’ represents ORs adjusted with age + sex, and age + sex + duration in years and age + sex, and age + sex + duration in years + ASIA scale respectively. ^α^ depicts the odds ratio with the female gender as a reference. ^β^ depicts the odds ratio with age < 25 years as a reference. ^¥^ depicts the odds ratio with SCI duration < 10 years. ^£^ depicts the odds ratio with ASIA scale ≤ B. $ depicts the independent variables one, two, and ≥ chronic complications have been checked in comparison to ones with no chronic complications.

**Appendix 3. Univariate and multivariate analysis for factors associated with the development of bacteriuria in the studied cohort.**

| Variables | Bacteriuria (univariate analysis) | | Bacteriuria (multi-variate analysis) | | | | | |
| --- | --- | --- | --- | --- | --- | --- | --- | --- |
|  | OR (95% CI) | P | Model ‘a’ | | Model ‘b’ | | Model ‘c’ | |
|  |  |  | OR (95% CI) | P | OR (95% CI) | P | OR (95% CI) | P |
| Male gender ^α^ | 0.9 (0.6 - 1.4) | 0.799 | 0.9 (0.6 – 1.4) * | 0.803 | 0.9 (0.6 – 1.4) * | 0.748 | 0.9 (0.6 -1.4) * | 0.712 |
| Age ≥ 25 years ^β^ | 0.8 (0.6 - 1.1) | 0.223 | 0.8 (0.6 -1.1) * | 0.213 | 0.9 (0.5 – 1.3) * | 0.667 | 0.9 (0.5 – 1.3) * | 0.66 |
| SCI duration ≥ 10 years^¥^ | 0.8 (0.6 - 1.2) | 0.462 | 0.9 (0.6 – 1.4) | 0.77 | - | - | 0.9 (0.6 – 1.4) * | 0.68 |
| ASIA scale ≥ C^£^ | 1.4 (1.0 - 2.1) | **0.038** | 1.4 (1.0 - 2.1) | 0.037 | 1.4 (1.0 - 2.1) | 0.041 | - | - |
| One chronic complication^$^ | 1.2 (0.9 -1.6) | 0.192 | 1.2 (0.9 -1.6) | 0.193 | 1.2 (0.9 -1.6) | 0.196 | 1.3 (0.9 – 1.7) | 0.096 |
| Two chronic complications^$^ | 1.0 (0.5 - 1.8) | 0.992 | 1.0 (0.5 - 1.8) | 0.979 | 1.0 (0.5 - 1.8) | 0.982 | 1.0 (0.6 - 1.9) | 0.853 |
| ≥Three complications^$^ | 0.8 (0.5 –1.4) | 0.608 | 0.8 (0.5 –1.5) | 0.464 | 0.8 (0.5 –1.5) | 0.474 | 0.8 (0.5 –1.5) | 0.644 |

ASIA: The American Spinal Injury Association (ASIA) Impairment Scale; CI: confidence interval; OR: odds ratio; SCI: spinal cord injury.

Note: The data has been generated by logistic regression analysis where pyuria and bacteriuria dare dependent variables. Model ‘a’, ‘b’, and ‘c’ represents ORs adjusted with age + sex, and age + sex + duration in years and age + sex, and age + sex + duration in years + ASIA scale respectively. ^α^ depicts the odds ratio with the female gender as a reference. ^β^ depicts the odds ratio with age < 25 years as a reference. ^¥^ depicts the odds ratio with SCI duration < 10 years. ^£^ depicts the odds ratio with ASIA scale ≤ B. ^$^ depicts the independent variables one, two, and ≥ chronic complications have been checked in comparison to ones with no chronic complications.
